# When It Hurts (and Helps) to Try: The Role of Effort in Language Learning

**DOI:** 10.1371/journal.pone.0101806

**Published:** 2014-07-21

**Authors:** Amy S. Finn, Taraz Lee, Allison Kraus, Carla L. Hudson Kam

**Affiliations:** 1 Department of Brain and Cognitive Sciences, Massachusetts Institute of Technology, Boston, Massachusetts, United States of America; 2 Department of Psychological and Brain Sciences, University of California Santa Barbara, Santa Barbara, California, United States of America; 3 Department of Psychology, Stanford University, Palo Alto, California, United States of America; 4 Department of Linguistics, University of British Columbia, Vancouver, British Columbia, Canada; University of Barcelona, Spain

## Abstract

Compared to children, adults are bad at learning language. This is counterintuitive; adults outperform children on most measures of cognition, especially those that involve effort (which continue to mature into early adulthood). The present study asks whether these mature effortful abilities interfere with language learning in adults and further, whether interference occurs equally for aspects of language that adults are good (word-segmentation) versus bad (grammar) at learning. Learners were exposed to an artificial language comprised of statistically defined words that belong to phonologically defined categories (grammar). Exposure occurred under passive or effortful conditions. Passive learners were told to listen while effortful learners were instructed to try to 1) learn the words, 2) learn the categories, or 3) learn the category-order. Effortful learners showed an advantage for learning words while passive learners showed an advantage for learning the categories. Effort can therefore hurt the learning of categories.

## Introduction

A great deal of research demonstrates that children surpass adults in their ultimate attainment of language [1]–[3]. This sensitive period for language learning poses a puzzle: why do children outperform adults in learning language but not countless other measures of learning and cognitive ability? While an explanation will likely include numerous factors (e.g., differences in existing knowledge, [4],[5]; entrenchment/over-learning [6]; neural plasticity [7]), a rather counter-intuitive explanation has been gaining attention recently: that adults' superior (domain-general) cognitive abilities interfere with learning certain aspects of language [2], [8]–[11]. However, little direct evidence is available to support this intriguing idea. We assess this hypothesis by directing adults' effort toward learning in an artificial language learning task, and ask whether trying to learn interferes with learning some types of linguistic information.

### The sensitive period for language

Although children are better language-learners, their advantage over adults is not universal. Adults learn many aspects of language more quickly [3]. However, over the longer term, few adults achieve native proficiency in speech production and perception [12], and—the focus of the present work—various aspects of grammar [2], [13] (i.e. categories of items and their category-based relationships to each other). For example, adult second-language (L2) learners are more likely than child L2 learners to endorse ungrammatical sentences. This is especially true for errors such as incorrectly placed determiners (e.g., a, the) or incorrect morphology (e.g. plural -s), but not errors of more basic overall word order (e.g., SVO ordering), or vocabulary [2], [14]. Older (i.e., adolescent or adult) L1 learners have also been shown to incorrectly endorse ungrammatical sentences [1], and produce more incorrect and “frozen” structures (whole phrases or sentences used in ways inconsistent with their internal structure) [2], [15] than younger L1 learners.

### The development of (domain-general) cognitive abilities

In contrast to language learning, adults show an advantage over children on many measures of cognitive ability that require sustained attention and effort. This protracted development is true for various conceptualizations of cognitive ability, including cognitive control [16], [17], working memory (WM; the ability to manipulate and hold information in mind [18], [19]), declarative memory [20], and endogenous attention [21]. Although these are distinct aspects of cognitive function, they are all attentionally demanding and recruit (to varying degrees) a similar suite of regions within prefrontal and parietal cortex [22]. As expected based on behavioral work characterizing the slow development of these cognitive abilities, these regions develop slowly [23]. In contrast, more automatic forms of learning (e.g., procedural or implicit; associated with the basal ganglia and cerebellum [24]) appear to develop more quickly [25], [26].

### Domain general abilities and language learning

Procedural learning is thought to be especially important for learning grammar [8], [27]. Several studies have shown that attentional processes and explicit forms of memory can interfere with procedural learning [28]–[30]. For example, when subjects were directed to try to learn the underlying structure of a stimulus (either the markov chain structure comprising an artificial grammar [29] or a highly complex alternating sequence of locations in a serial reaction time task [30]) the basic instruction to try to learn led to poorer learning as compared to participants not told to try. Interestingly, in the case of artificial grammar learning, if subjects were first explicitly taught what the structure of a markov chain grammar looks like, instructing subjects to try improved learning. The explicit teaching appears to modify the effect of effort by constraining the hypothesis space for learners. The subjects were directed to look for the right kind of structure (without being told what the particular features of that structure were). Subjects told to learn the grammar but without the teaching were searching in a much larger hypothesis space, and so were much less likely to find the right answer and much more likely to find an incorrect one. It is this last part that distinguishes them from the subjects who weren't trying to figure out the grammar at all. Thus, it appears that although trying can impair performance, it can also be beneficial, if the resources are directed properly and incorrect hypotheses need not be tested.

Effort requires attention, or the direction of mental resources toward a particular goal. Attentional learning systems are known to have capacity limitations [31]. Searching a limitless hypothesis space or holding multiple complex possibilities in mind is not possible given these limitations. Therefore, trying to learn structure that is complex and more likely to exceed these capacity limitations is difficult, if not impossible, using an attentional learning system. Indeed, the procedural memory system may be better suited for learning this kind of information [27]. In support of these ideas, one study looking at the impact of declarative memory on procedural learning showed that the negative impact on procedural learning was greater for those who had greater mnemonic capacities [28]. Thus, effortful mnemonic systems that require attention may not be well suited to learning complex and irregular structure, such as that present in grammar. From the perspective of language learning, this would impede the acquisition of grammatical aspects of the language, but not simple word segmentation or basic word order. By implication, a learner with less attentional capacity, especially relative to their procedural resources, would have less interference and better learning outcomes. Thus, children *could* simply be better built to learn grammar.

This view predicts that effort should have a measurable effect on grammar learning in adults. While no one has explored the role of effort with sensitive period effects in mind, studies have explored the role of attention in word segmentation. The use of transitional probability (TP, the probability of *Y*|*X*  =  (frequency of *XY*)/(frequency of *X*)) information for word segmentation, or “statistical learning” is thought to occur without attention —or procedurally[32]. Two lines of evidence suggest that learning in these kinds of experiments is largely procedural. First, young infants and other mammals (who are less likely to be consciously exerting attentional resources) can perform word segmentation using TPs [33]–[35]. Second, both children and adults can do this (use TPs for word segmentation) when engaged in a non-attentionally taxing alternate cover task with the stimuli playing in the background [36]. They cannot be computing TPs because they are actively trying to, given that they are unaware that they will later be tested on their knowledge of aspects of the stimuli involving TPs. Given this, one might think that attention is not helpful for learning aspects of language involving these kinds of computations.

Although the studies just discussed show that attention is not necessary, other studies show that it can be beneficial for statistical learning. For instance, directing adults' attention towards a distractor task can impair learning [37]. Likewise, directing adults' attention to a subset of stimuli results in successful segmentation of the attended, but not unattended, items [38]. Moreover, manipulating attention has a greater impact on the segmentation when words have lower (less predictive) versus higher TPs [39]. In sum, when attention is directed toward the stimulus, learning is better than when it is not. When it is taxed for another purpose and turned away from the to-be-learned stimulus, learning is impaired, and, when attention is not taxed, but another non-taxing cover task is used, learning occurs as normal. These studies therefore show that learning is improved with attention. This stands in contrast to the above-cited studies on other forms of procedural learning where effort appears to harm the learning of a markov chain grammar or a complex alternating sequence. The difference, we suggest, is in the nature of the material to be learned.

Word segmentation involves the learning or extraction of specific items and the relationships between them. Attention is beneficial in this kind of simple task. As discussed above, however, while aspects of complex patterns can be learned without effort and attention, when effort is directed at them it can be harmful if the learner is left to search a limitless hypothesis space and left with no information about what kind of pattern they are looking for ([30], [40] cf. [41]).

We therefore ask whether effort facilitates or impairs adults' learning of certain aspects of language that 1) are learned similarly by children and adults, and 2) that adults are known to have difficulty learning. Specifically, we compared performance on word segmentation and category/category relationship learning under different attentional conditions. Word segmentation may not seem like a natural contrast to grammar. From the perspective of the sensitive period however, word segmentation ability is relatively age-invariant [36] in the absence of attention, and is item-based, making it ideal given our hypothesis. (Adults may also learn word meanings differently than children [42] and so vocabulary learning might not be as age-invariant as has been previously assumed.) If our ideas are correct, effort should facilitate word-segmentation, but harm the learning of novel grammatical categories and their behavior.

## Experiment 1

Although studies have demonstrated that both word segmentation and grammar learning can occur based purely on distributional information in artificial languages ([43]–[46] [even simultaneously, [47]]), to our knowledge, no one has compared learning of the two kinds of information, particularly from the perspective of understanding the sensitive period for language acquisition. Therefore in the first experiment, adult learners were exposed to a continuous speech stream containing TP-defined words organized into categories, which occurred in a consistent order. We assessed whether participants had 1) segmented the words, and 2) learned the categories. Learners' attention was not directed towards the stimulus allowing us to assess the outcome of implicit learning.

### Method

#### Participants

Twenty-two native English-speaking undergraduates (mean age: 21.67 years, standard deviation: 3.9 years; 81% female) at the University of California, Berkeley participated for course credit. Written consent was obtained from these and all participants in the study. The institutional review board at the University of California, Berkeley approved this study.

#### Stimuli

**Figure 1:**
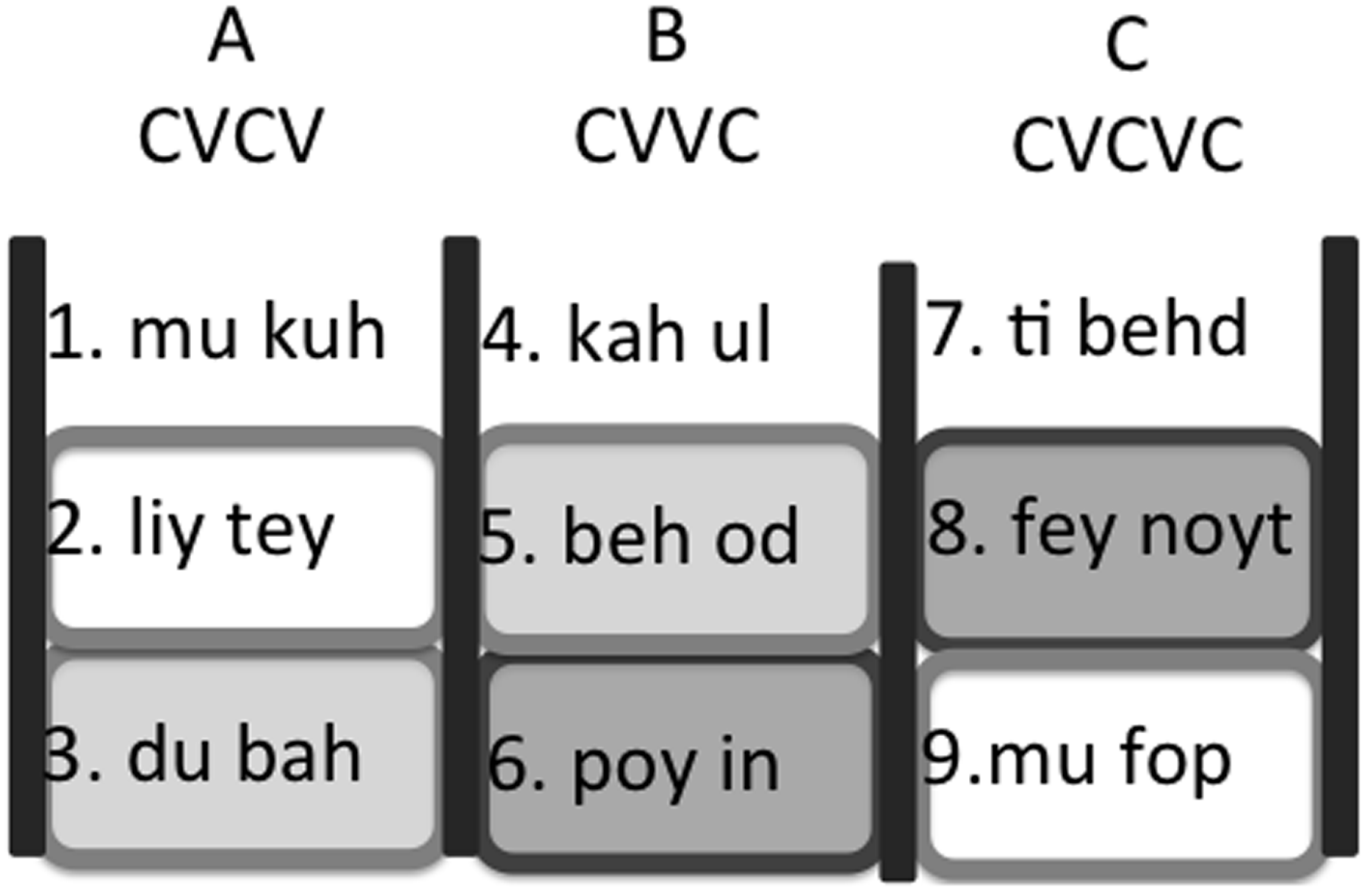
Structure of the Artificial Language. Similarly shaded pairs were withheld from the stimulus presentation.

The exposure speech stream lasted just under 10 minutes and was constructed using nine two-syllable words strung together without pauses or other acoustic cues to word boundaries. Each word belonged to one of three categories (A, B, C). Category members shared a phonological structure as well as distribution (Figure 1). All words (and syllables) were consistent with English phonotactics but were not meaningful words in English.

A words were followed by B words, which were followed by C words, which were then followed by A words, and so on. Each word occurred 45 times. Since different TPs have been shown to be differentially affected by attention [39], TP variability was created by constraining the presentation order such that one word from each category never followed another particular word from the preceding category. Words were otherwise presented randomly. TPs were 1.0 for word internal syllable transitions and ranged from .33 to .5 across word boundaries. However, category-to-category TPs were 1.0. Thus, word order was much more predictable at the level of categories than at the level of syllables or words.

This kind of shared phonological structure mimics tendencies in real languages in exactly the kinds of categories that adults have difficulty learning (e.g., noun classes). It is also known to assist adult learners in acquiring categories in similar experiments, as compared to the use of purely distributional information [48]. Thus, the phonological cue should make category learning (and therefore ordering, since you need the category to learn its ordering) easier. Importantly, this is the very kind of abstract category structure that adults have difficulty learning, and so might be susceptible to the effort effect we are examining. An example stimulus stream is as follows: *…mukuhbehodfeynoytdubah kahulmufop…*

The artificial language stimulus stream and test items were generated with text-to-speech software that uses terminal analog formant synthesis (and not pre-recorded di-phones) [49]. This was chosen over natural speech (and diphone based methods) to eliminate segmentation cues that were not experimentally relevant (including those that indicate a segment's location in the syllable; i.e., release bursts). All of the vowels in all of the stimuli and tests were the same length (170 ms) and consonants ranged from 60 ms to 140 ms (but were always the same for that phone regardless of their location). These lengths were automatically generated using the average speaking rate setting in the software.

#### Tests

After exposure, participants completed two forced choice tests: 1) a word-level test in which they were asked which of two words was more likely to belong in the language they just listened to, and 2) a sentence-level test in which they were asked which sentence was more likely to belong in the language they just listened to. They always completed the word-level test first and test items for each test were randomized separately for each subject. In these 2 tests, there were three test types of interest: *word segmentation, order* and *category structure*. All word segmentation items occurred in the first, word-level, test and all of the order and category structure occurred in the second, sentence-level, test.

The word segmentation test assessed whether participants had extracted the words (defined by TPs) from the speech stream. Participants were asked to choose between a word (word-internal syllable TPs = 1.0) and either a non-word (the first syllable of one word and the second syllable from a different word, e.g. mu-tey) or part-word (the second syllable from one word paired with the first syllable from a word in the adjacent category, e.g., kuh-poy). TPs for non-words were always 0 and part words were .33 or .5. There were 9 of each type (18 total).

In the order test, participants were asked to compare strings that followed the correct order with strings that did not (the lack of pauses combined with the TP structure at the category-level means that there is no real start or end to a multi-item string; C–A–B is just as correct as A–B–C). Test items comprised two strings with the same words and same first word, but in different orders, e.g. A(mu kuh)-B(kah ul)-C(ti behd) vs. A(mu kuh)-C(ti behd)-B(kah ul). The across-word TPs for the ungrammatical strings was 0–0 both at the level of the syllable and at the level of the category (C never comes after A, nor B after C). (From here forward test items are described only in terms of word- or syllable-level TPs, since the category-level TPs are always 1–1 for grammatical and 0–0 for ungrammatical strings.) The across-word TPs in the grammatical strings was either .33–.33, .5–.5, or mixed (i.e., .5–.33). There were 21 test items.

The category structure test probed learning with the use of novel words. 9 novel items (3/category) that followed the phonological structure of the relevant category (category-congruent) and 4 novel items that did not fit into any category (e.g. CVCCV, VCCV; category-incongruent) were generated. These novel words were put into grammatical and ungrammatical locations, in strings with and without TP cues, creating 3 subtypes: *Novel-with-TP*, *Novel-no-TP* and *Novel-good-vs.-bad*. In novel-with-TP items, a category-congruent novel word was placed in either the correct or incorrect order and TP cues were present in the grammatical string (i.e., A-B-C_novel_ vs. A-C_novel_-B; TP = .5 or.33–0, vs. 0–0). These test items indicate how learners deal with novelty when a distributional cue is present. In Novel-no-TP items, learners were asked to compare strings with a category-congruent novel word in one of two places (as before), but with no TP cue (A-B_novel_-C vs. A-C-B_novel_; TP = 0–0 vs. 0–0). Finally, Novel-good-vs.-bad items contained strings with category-congruent and -incongruent novel words in the same place and no TP information (A-B_novel-congurent_-C vs. A-B_novel-incongruent_-C; TP = 0–0 for both). Correct performance on the latter two types requires knowledge of which phonological structures belong in which relative positions. Table 1 lists the structure of each of the test items by category and sub-type. See Methods S1 for further details on the methods and stimuli.

**Table 1:** Test Types and Examples.

***Word Segmentation***		
*Type (TP)*	*Correct*	*Foil*
1 vs 0	mu kuh	mu tey
1 vs .33	mu kuh	kuh beh
1 vs .5	du bah	bah poy
***Order***		
*Type (TP)*	*Correct*	*Foil*
.33 vs 0	A(mu kuh)-B(kah ul)-C(ti behd)	A(mu kuh)-C(ti behd)-B(kah ul)
.5 vs 0	A(du bah)-B(poy in)-C(ti behd)	A(du bah)-C(ti behd)-B(poy in)
mix vs .0	A(du bah)-B(kah ul)-C(ti behd)	A(du bah)-C(ti behd)-B(kah ul)
***Category Structure***		
*Type (TP)*	*Correct*	*Foil*
Novel with TP	A-B-C_novel_	A-C_novel_-B
Novel no TP	A-B_novel_-C	A-C-B_novel_
Novel Good vs Bad	A-B_novel-congurent_-C	A-B_novel-incongruent_-C

#### Procedure

Exposure and testing were conducted individually. Participants were told to listen to an artificial language and neither over-think nor ignore it. To encourage this and following the procedure in the original paper showing that statistical learning can occur incidentally [36], participants were asked to color during exposure. After exposure, participants completed the forced choice tests. All tests were administered on a computer using E-Prime software [50].

## Results and Discussion

### Word Segmentation

**Figure 2:**
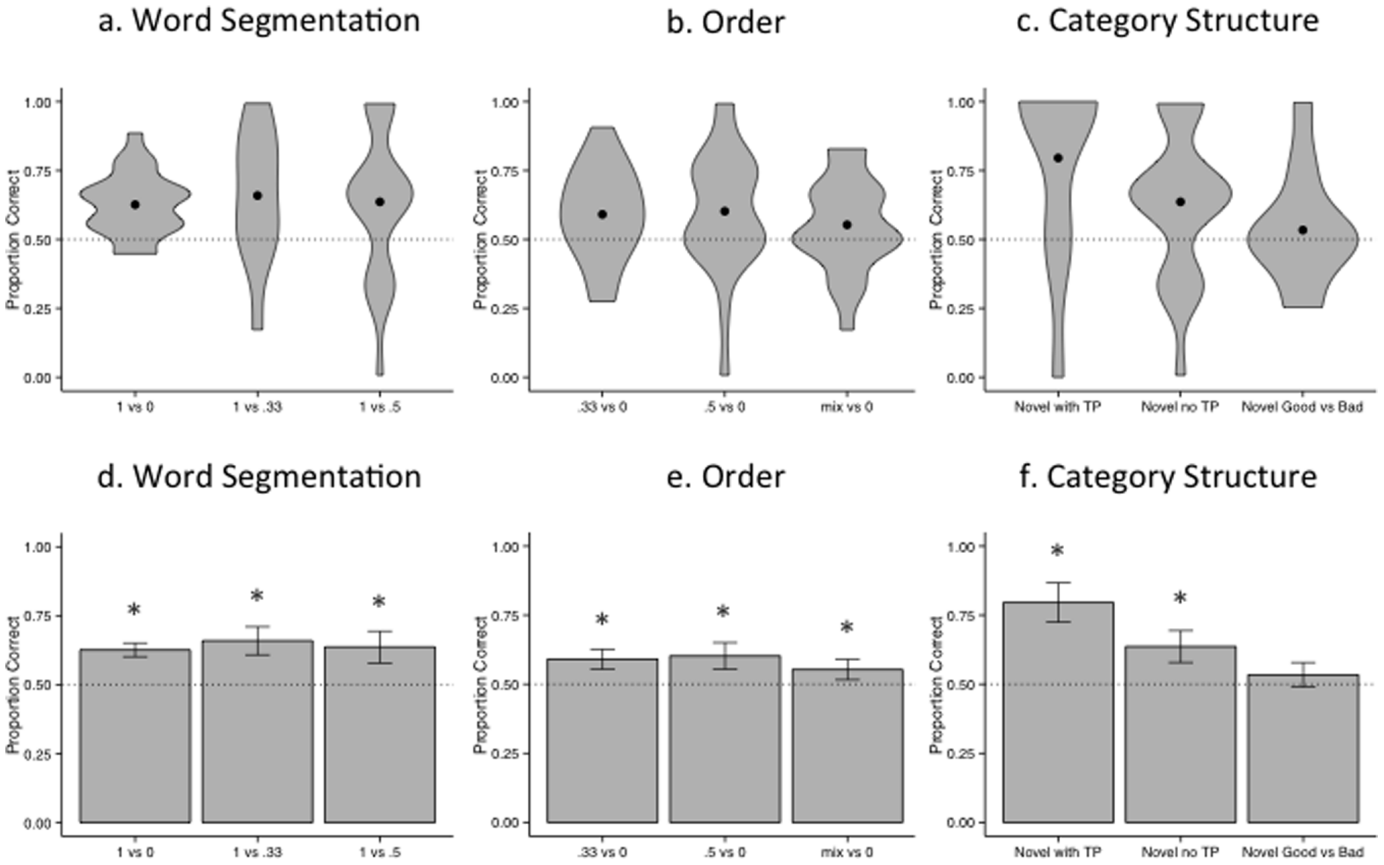
Experiment 1. Performance on word segmentation (a,d), order (b,e) and (c,f) category-structure sub tests. Violin plots (a–c) depict the minimum (bottom of shape) and maximum (top of shape) observed values. Black circles indicate the group mean, and width indicates the probability density of the value on the corresponding y axis. Chance performance is indicated with the dotted line. Bar graphs (d–f) depict the group mean. Error bars reflect standard error of the group mean and chance performance is indicated with the dotted line.

Performance on the word-segmentation items is shown in Figure 2a. A repeated measures analysis of variance (ANOVA) with performance on the three word segmentation sub-tests (TP 1 vs. 0, 1 vs. 5, 1 vs. .33) as a within-subjects factor reveals that performance does not differ across the sub-tests (*F*(2,42) = .123, *p* = .884, *η_p_^2^* = .006). Therefore, more fine-grained TP comparisons (.5/.33 vs. 1) are not more difficult than grosser comparisons (0 vs. 1). Importantly, performance is significantly better than chance (*t*(21) = 6.03, *p*<.001, *d* = 1.3), demonstrating that learning was successful.

### Order

A repeated measures ANOVA with performance on the three order sub-tests (TP .33 vs. 0, .5 vs. 0, mixed (.33or.5) vs. 0) as a within-subjects factor reveals that performance does not differ across the sub-tests of order (*F*(2,42) = .402, *p* = .672, *η_p_^2^* = .019), shown in Figure 2b. More fine-grained TP comparisons (.5/.5 vs. 1) are not more difficult than grosser comparisons (.33/.33 vs. 1 or mixed vs. 1). Performance is again above chance (*t*(21) = 3.61, *p* = .002, *d* = .75), demonstrating that participants learned the categories and their relative ordering.

### Category Structure

A repeated measures ANOVA with performance on the three category structure sub-tests (novel with a TP cue, novel without a TP cue, and novel good versus bad items) as a within-subjects factor reveals that performance differs across sub-tests of category structure (*F*(2,42) = 5.79, *p* = .006, *η_p_^2^* = .216), see Figure 2c. To understand which sub-types were learned, we compared performance on each sub-test to chance individually, revealing successful learning when novel (category-congruent) items are presented both with (*t*(21) = 2.37, *p* = .028, *d* = .504) and without (*t*(21) = 4.16, *p*<.001, *d* = .887) a TP cue. Performance on this second measure (without a TP cue) indicates participants have learned the abstract phonologically-defined category structure, as well as how the categories are ordered, and that they do not need familiar sequences of words to distinguish a grammatical from an ungrammatical string. However, learners are not significantly different from chance on items that compare category-congruent with category-incongruent novel words (*t*(21) = .767, *p* = .451, *d* = .165), suggesting that this knowledge is not robust enough to rule out sequences about which they have no information (novel types).

## Experiment 2

Experiment 1 established that individuals can segment word-like units, extract information about the order of categories, and learn something about the phonological structure of said categories under typical implicit learning conditions. In the next experiment we explore effortful learning.

### Method

#### Participants

Sixty-six native-English speaking undergraduates (mean age: 21.66 years, standard deviation: 3.85 years; 70% female) at the University of California, Berkeley participated for course credit. There were no significant differences across learning conditions for working memory (*F*(2,61) = .002, *p* = .998), age *F*(2,61) = .272, *p* = .763), number of females *F*(2,61) = .538, *p* = .586), or number of bilingual (*F*(2,61) = 1.56, *p* = .218) participants. Working memory ability was measured via the reading span task [51], [52].

*Stimuli & Test items*

All stimuli and tests were the same as Experiment 1. As in Experiment 1, all tests were administered using E-prime software for the first and second conditions (described below, effort towards words and effort towards kinds respectively) and using Psychopy software [53] for the final condition (effort toward order, described below).

#### Procedure

The procedure mimicked Experiment 1 except that, prior to exposure: one-third (22) of the participants were told to try to learn the nine words present in the language, another third were told that there were 3 “kinds or categories of words” in the language and that they were to try and determine what these 3 categories were, the final third were told that there were 3 categories of words that follow each other in a specific order and to try and determine what the order is. All were warned that there were no pauses between the words. To ensure continued attention, participants were given a task during exposure. They were asked to press one of two buttons over the course of learning to indicate their knowledge of the aspect of the language they were trying to learn. In the event that they had an idea about what a word (or kind/category, or category-order) was, they were asked to press the white button. They could do this as many times as they like, but were asked to do this each time they had a strong idea or hypothesis about what one might be. In addition, they were asked to press a red button whenever they decided that they had learned a word (or kind/category, or category-order). They were asked to be more conservative with this button, but they were also told that it was fine to press this more than the number of items that they were trying to learn (so, e.g., more than 9 times in the word condition) if they changed their minds later during exposure. This manipulation had the effect of focusing subjects on the learning task at hand. Indeed, many indicated that pressing the button was quite rewarding during an otherwise rather boring task.

## Results and Discussion

Because we are interested in the effects of effort on learning, we compare the performance of the 3 effort conditions to that of the no-effort condition (Experiment 1) on each of the tasks separately. Our prediction is that effort should hurt grammar learning, but not word segmentation, relative to no effort. We did not predict that effort should hurt grammar learning relative to word segmentation.

### Word Segmentation

**Figure 3:**
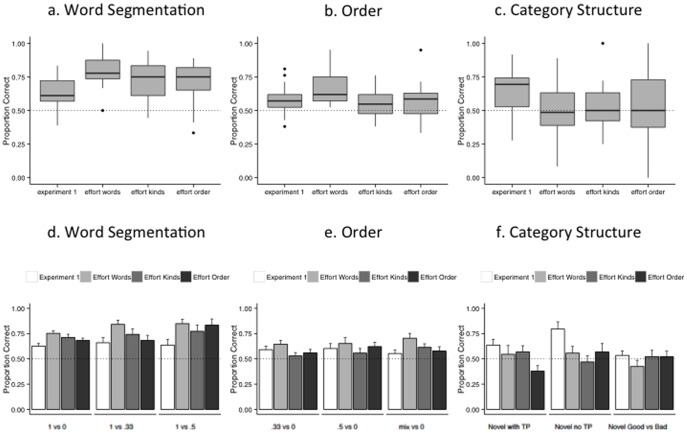
Group differences. Performance on word segmentation (a,d), order (b,e) and (c,f) category-structure sub tests. Performance on word segmentation (a,d), order (b,e) and (c,f) category-structure sub tests. Box plots (a–c) depict the median (middle line), upper quartiel (top of box), lower quartile (botton of box), maximum value (top whisker, excluding outliners), and minimum value (bottom whisker excluding outliers); outliers are each indicated with circles. Chance performance is indicated with the dotted line. Bar graphs (d–f) depict the group mean. Error bars reflect standard error of the group mean and chance performance is indicated with the dotted line.

Figure 3a shows performance across groups and Figure 3d shows performance for each of the groups on each of the sub-tests. A repeated measures ANOVA with performance on the three word segmentation sub-tests (TP 1 vs. 0, 1 vs. 5, 1 vs. .33) as a within-subjects factor and learning condition (group) as the between-subjects factor reveals a main effect of group (*F*(3,84) = 5.317, *p* = .002, *η_p_^2^* = .160) and sub-test (*F*(2,168) = 3.625, *p* = .029, *η_p_^2^* = .041), but no group by sub-test interaction (*F*(6,168) = 1.024, *p* = .411, *η_p_^2^* = .035). All groups performed better than chance on this measure (no-effort: *t*(21) = 6.03, *p*<.001, *d* = 1.29; effort-words: *t*(21) = 12.65, *p*<.001, *d* = 2.70; effort-kinds: *t*(21) = 7.18, *p*<.001, *d* = 1.53; effort-order: *t*(21) = 6.67, *p*<.001, *d* = 1.422). Performance was better for the effort groups relative to the no-effort group (Dunnett's: word vs. no-effort *p*<.001, effort-kinds vs. no-effort *p* = .029, effort-order vs. no-effort *p* = .049). Consistent with previous research, trying to segment words facilitates segmentation. Indeed, trying to learn anything about the speech stream facilitates segmentation. As in Experiment 1, more fine-grained TP comparisons (.5 and .33 vs. 1) are not more difficult than grosser comparisons (0 vs. 1); in fact, the opposite may be true; collapsing across groups, performance is better for .5 TP items than it is for 0 (*t*(87) = −2.64, *p* = .010, *d* = .3; Bonferroni corrected α = .017) but there were no differences between the other types (.33 vs. 0 (*t*(87) = −1.571, *p* = .120 *d* = .18; .33 vs. .5 (*t*(87) = −1.238, *p* = .219, *d* = .13).

### Order

Figure 3b shows performance across groups and Figure 3e shows performance for each of the groups on each of the sub-tests. A repeated measures ANOVA with performance on the three order sub-tests (TP .33 vs. 0, .5 vs. 0, mixed (.33or.5) vs. 0) as a within-subjects factor and learning condition (group) as the between-subjects factor reveals a main effect of group (*F*(3,84) = 3.22, *p* = 027, *η_p_^2^* = .103), but not sub-test (*F*(2,168) = .668, *p* = .514, *η_p_^2^* = .008), and no group by sub-test interaction (*F*(6,168) = .677, *p* = .669, *η_p_^2^* = .024; Figure 3b). All groups performed better than chance (one-sample, two-tailed t-tests: no-effort: *t*(21) = 3.61, *p* = .002, *d* = .77; effort-words: *t*(21) = 6.23, *p*<.001, *d* = 1.33; effort-kinds: *t*(21) = 2.98, *p* = .007, *d* = .64; effort-order: *t*(21) = 2.81, *p* = .011, *d* = .60). Performance was better for the effort-word group relative to the no-effort group (Dunnett's *p* = .025), but not for the other effort groups as compared to the no-effort (Dunnett's: effort-kinds vs. no-effort, *p* = .880; effort-order vs. no-effort, *p* = .708) Trying to learn the words, but not the categories or the order of categories, appears to facilitate learning of order.

### Category Structure

Figure 3c shows performance across groups and Figure 3f shows performance for each of the groups on each of the sub-tests. A repeated measures ANOVA with performance on the three category structure sub-tests (novel with TP, novel no TP, and novel good versus bad) as a within-subjects factor and learning condition (group) as the between-subjects factor reveals a main effect of group (*F*(3,84) = 3.74, *p* = .014, *η_p_^2^* = .118), and of sub-test (*F*(2,168) = 4.33, *p* = .015, *η_p_^2^* = .049), but no group by sub-type interaction (*F*(6,168) = 1.51, *p* = .179, *η_p_^2^* = .051; Figure 3c). Here, only the no-effort group was significantly better than chance (*t*(21) = 4.08, *p* = .001, *d* = .87; effort-words: *t*(21) = .22, *p* = .825, *d* = .05; effort-kinds: *t*(21) = .55, *p* = .588, *d* = .12; effort-order: *t*(21) = .43, *p* = .672, *d* = .09), and just on 2 of the 3 sub-tests: novel-with-TP (*t*(21) = 2.37, *p* = .028, *d* = .5) and novel-no-TP (*t*(21) = 4.16, *p*<.001, *d* = .89). The no-effort group outperformed all effort groups on this measure (Dunnett's: no-effort vs. effort-word, *p* = .013, no-effort vs. effort-kinds, *p* = .022, no-effort vs. effort-order, *p* = .005). Across groups, performance is better for novel-no-TP than novel-with-TP (*t*(87) = 2.95, *p* = .004, *d* = .32, Bonferroni corrected α = .017), but not for other sub-tests (novel-with-TP vs. novel-good-vs-bad: *t*(87) = 1.651, *p* = .102, *d* = .18; novel-good-vs-bad vs. novel-no-TP: *t*(87) = 1.27, *p* = .202, *d* = .14). These data show that the no-effort learners outperform the other groups, a result that is most notable for the novel-no-TP items (Figure 3). Effort therefore appears to hurt the learning of at least some kinds of category structures. (See Figures S1 and S2 for further analyses.)

## General Discussion

The present study investigates whether mature effortful processing abilities interfere with language learning in adults and whether this is differentially true for aspects of language that adults are better or worse at learning (e.g., word segmentation vs. grammar). We found that directing effort toward the stimulus helps for word segmentation but hurts the learning of category structure, and has a mixed effect (although mostly null) on the learning of the ordering of the categories. This represents the first experimental evidence that differences in effort are related to learning outcomes akin to what is observed in nature: adults' general superior learning ability, but inferior ability to learn grammar.

While all groups of learners were able to segment the words (performance was above chance), the effort groups segmented better than the no-effort group. Effort therefore facilitates word segmentation. This is in line with work showing that attention is important for word segmentation.

In contrast, effort appears to hinder the learning of the phonological organization of the categories; effort led to chance performance on tests of category structure, despite the fact that these same participants were better at segmenting the words. This is true even when learners were told explicitly about the existence of categories. However, learners who were told nothing were better than chance on the novel-with-TP and novel-no-TP sub-tests. Moreover, these no-effort learners were significantly better than the effortful groups. This pattern of results has direct implications for understanding why children are better at learning grammar, or rather, for understanding why adults are worse. This is consistent with the idea that that adults' difficulty has to do with their superior ability to exert effort.

For word segmentation, we were not expecting to find that that performance, across groups, for one of the two fine-grained TP comparisons would be better than the grosser comparison (TP = 0 vs 1). Recall that in the TP 0 vs. 1, a syllable from another word is added to the same first syllable, so one would compare the word *mu-kuh* with *mu-bah*. In the finer-grained comparison, one always compares a word like *mu-kuh* and takes the same second syllable (in this case *kuh*) and combines it with something that follows it only sometimes (i.e., *kuh-poy*). It could be that it is easier to make the comparison when the second syllable of the word becomes the onset, although this should not be obvious to the subject as TP is the only segmentation cue. It could also be that making comparisons that have the same onset (i.e., *mu*) is inherently more difficult. Speculatively, it could be the case that this requires more explicit comparison, which would not be beneficial in a forced choice test. Still, if it were about grosser versus finer, we would expect subjects to be better at both .33 and .5 vs. 1 than 0 vs. 1 and this is not the case. Likewise if it were graded in this direction such that only more exaggerated differences were observed, one would expect that participants would be best on the .33 vs. 1, not .5 vs. 1. This subtle difference therefore needs to be explored (and all of the above noted possibilities) in a more direct and separate experiment designed to test these possibilities.

Note also, that we did not find that effort had a differential effect on higher vs. lower TPs. Previous work examined differing TP values within words [39], and we examined these in the incorrect test options. However, we discovered a new pattern: when the TPs should have helped, they often did not. On the category-structure test, performance was better when the string contained no word-level TP information. Similarly, when the foil in the word segmentation test contained a TP of .5, participants performed better than when it was 0. We do not wish to make a great deal of these results, as they are unpredicted, however, it is intriguing that they both go in the same direction.

Interestingly, performance on the order test, designed to mimic the learning of broad word order patterns—something adults are good at learning—was neither consistently improved nor hindered by effort. That is, focusing on words (but not kinds or the ordering of categories) led to better performance on this test. Why would focusing on words be especially helpful? It could be that focusing on words (and not necessarily learning them well since that was true of all of the effort learning groups), could be an important step in learning about how those units are ordered. This is likely the case since the test of order in this experiment requires no abstraction. All learning of order is taken directly from exposure and so it makes sense that focusing on the units could help in learning about how they go together. However, one would also expect there to be better word segmentation (learning of the units) with effort towards words as compared to effort towards order or kinds if this were the case, which we did not find (all effort groups segmented the words very well). Future work should explore these relationships in greater detail. It is also noteworthy that all participants who did not acquire the phonological structure underlying the categories (all of the attention learners who showed chance performance on the category structure test), still performed significantly above chance on the order measure. This suggests that they were doing so via different information. It also demonstrates that there are different routes to what appears to be similar levels of performance.

Despite these results, attention can be beneficial for language learning. However, this is generally observed in the context of explicit learning environments, i.e., the classroom, which specifically take advantage of adults' attention-based learning abilities [54], [55]. More naturalistic input situations are very different: a learner does not know a priori what they need to know, or what specific patterns they need to find, even when (as in our study) they know that there are patterns to be found. Thus, one way adults might overcome the disadvantages of having better cognitive capacities that impede the implicit learning of language patterns is to use those same cognitive capacities to learn language in a different way.

Our pattern of data is an interesting contrast to what is observed in child first language-learners where language-learning success in one area is typically related to learning success in other later-learned areas [56]–[59]. Given this, why are good learners in our sample not good learners across the board? One possibility could be that adults learn the words too well; they learn each of the tokens, but fail to learn the internal structure of the tokens and then therefore fail to generalize and apply this knowledge in novel circumstances [60], [61]. Much follow-up work is required to know whether this is the case. It will be especially important for future work to explore the possible role of sleep in increasing the learning of categories such as these, which require abstraction beyond the specific word forms. Indeed, work in infants suggests that sleep might be beneficial for this kind of non-veridical learning [62].

It is important to note that language learning in the real world is much more complicated than it is in an artificial language experiment such as this. This, however, does not detract from our findings. Whether or not the effect of effort on learning is a major source of differences between child and adult language learners (we have explored other potential contributions to adult difficulties with language learning [4], [63]), it remains an interesting finding with respect to understanding the way that basic cognitive processes (such as attention and learning) interact with each other to sometimes produce different learning outcomes. And while our results may at first seem counterintuitive, when viewed through the lens of what is known about the operation of the underlying learning systems, they are actually quite predictable. One of the main points worth taking from our results is that learning, indeed any human behavior, needs to be examined in less simplistic ways, as outcomes are almost always going to result from multiple interacting factors, not just the operation of a single system in an unchanging environment. This work is a small step in that direction.

Having established that effort interferes with the learning of phonological patterns (category structure), but not distributional ones (word segmentation and order) in an artificial language, this study 1) replicates (for the first time) in the lab what is observed about adult language learning in nature and 2) opens a door (long shut) for further more detailed exploration about *why* adults have difficulty learning some, but not all, aspects of language. The various cognitive functions involved in effort and their possible contributions to this effect will need to be fleshed out. For instance, effort allows a learner to 1) hold some, but not all, of the input they are exposed to in mind (WM), and 2) to make explicit hypotheses about the relationship between items. Given known capacity limitations [31], effortfully holding things in mind is not likely to be the best way to learn complex information akin to what is present in grammar. Moreover, having the wrong explicit hypothesis can harm learning [29]. The present data therefore clearly show that, when it comes to learning language, trying is not always best.

## Supporting Information

Figure S1 **Average performance on each word in Experiment 1 (a) and Experiments 1 and 2 (b).** Error bars reflect standard error of the mean. The dotted line reflects chance performance.
(TIFF)

Figure S2 **The number of words (a) and syllables (b) plotted against performance on the word segmentation test.** Data are reported across all four groups of learners.
(TIFF)

Methods S1 (DOCX)
