# Supplementary material for: When It Hurts (and Helps) to Try: The Role of Effort in Language Learning
Source: PLoS One. 2014 Jul 21;9(7):e101806. doi: 10.1371/journal.pone.0101806 (PMC4105409; doi:10.1371/journal.pone.0101806)
Supplement: Methods S1 — (DOCX) [file pone.0101806.s003.docx]

*Methods S1*

*Stimuli:* The unsegmented speech stream, generated with the text-to-speech program SoftVoice [[1](#_ENREF_1)], lasted exactly 9 minutes, 37 seconds. The synthesizer produced syllables with a monotonic **F**0 (fundamental frequency) of 83.62 Hz. All vowels were matched for length and there were no coarticulation effects.

*Procedure:* Participants completed the word segmentation test prior to the category order and category structure tests (which were completed together as one test). Within each test (word segmentation, order and category structure), items of all sub-types were presented randomly on a subject-by-subject basis using E-prime software [[2](#_ENREF_2)]. The two items (either two words or two phrases) were presented one after another with a 700ms pause in between, and participants indicated their selection using a button press. Participants were told that they would listen to pairs of possible words (or sentences) and were asked to “indicate which is more likely to have belonged in the language” they were exposed to. They were encouraged to make their best guess if unsure and were reminded of these same instructions prior to the second test.

The final, declarative memory test, asked subjects 1) to produce the words they had heard, 2) how many words they thought were in a sentence, and 3) how many different kinds of words they thought were in the language. Responses were recorded (on paper) by the experimenter. For (1), productions were scored as either correct (it was exactly the word) or incorrect. We also noted the total number of syllables each participant correctly produced (regardless of whether they were produced in perfectly correct words).

*Test items (novel)*: Novel words were generated for the final test. The same set of consonants and vowels that were used to generate the exposure words were used to ensure that treatment of the words was due to the rules governing their phonological structure (and not reactions to novel sounds). Nine of these novel words were category-congruent (i.e., according with a phonological structure that occurred in the input) and 4 were category-incongruent (i.e., they had a novel phonological structure). The nine correct words were: category A: mukuh, litytey, dubah; category B: kahul, behod, poyin; category C: tibehd, feynoyt, mufop. The four incorrect words were: neytlah, puhnmu (CVCCV); and obtoy, iybdu (VCCV). Importantly, the category-incongruent words were comprised of the same consonants and vowels as the category-congruent novel words they were directly compared to. For example, the novel (category-congruent) word following category B structure /bɪʌt/ was compared with the category-incongruent novel word /ɪbtʌ/. Each novel item contained exactly the same phones, they were simply arranged in a different way. Both items were flanked by exactly the same words in the test phrase.

*Results*

*Item Analysis*

As can be seen in Figure S1, not all of the words were endorsed more often than chance by participants in Experiment 1. One sample t-tests (one-tailed) reveal that the words mukuh (*t*=1.667, *p*=.055) dubah (*t*=2.027, *p*=.028), kahul (*t*=4.948, *p*<.001), behod (*t*=1.555, *p*=.067) and feynoyt (*t*=6.062, *p*<.001) are endorsed more than foils significantly more often than chance, while the words liytey (*t*=.253, *p*=.402), poyin (*t*=.901, *p*=.189), tibehd (*t*=1.073, *p*=.148) and mufop (*t*=.326, *p*=.374) are not (although all of the means are numerically greater than chance). Recall that each item is tested only 2 times for each participant, so these data are rather noisy. If instead we look at the data for participants in both experiments, thereby increasing the sample size , all items are endorsed significantly more often than chance: One sample t-tests (one-tailed) mukuh (*t*=3.393, *p*<.001), liytey (*t*=3.574, *p*<.001), dubah (*t*=8.74, *p*<.001), kahul (*t*=9.499, *p*<.001), behod (*t*=8.274, *p*<.001), poyin (*t*=3.706, *p*<.001), tibehd (*t*=6.622, *p*<.001), feynoyt (*t*=8.651, *p*<.001) and mufop (*t*=5.422, *p*<.001).

*Declarative Knowledge*

Interesting group differences emerged in the declarative knowledge participants had about the language. Both number of syllables and number of words produced correctly showed an effect of learning conditions, with syllables being significantly different (*F*(3,59)=3.66, *p*=.017, *η_p_^2^*=.157) and words going slightly in that direction (*F*(3,59)=1.78, *p*=.168, *η_p_^2^*=.081),^1^. Post-hoc comparisons reveal that no-effort learners produced significantly fewer correct syllables than the effort-words (Dunnett’s *p*=.028) and effort-order (Dunnett’s *p*=.020) groups, but not the effort-kind group (although the means are in the correct direction: mean number correctly produced no-effort=1.7; mean effort-kinds=2.3 ; Dennet’s *p*=.357). Thus, compared to at least 2 of the 3 effort groups, the no-effort group had less declarative knowledge of the words in the artificial language, something we might expect given their poorer performance on the word segmentation test. Interestingly, group differences were not observed for other, less-specific, declarative knowledge about the language. Groups did not differ from one another in the number of words they thought were in a sentence (mean= 2.45) or how many different kinds of words they thought there were (mean=1.36; this question was not asked of the 2 groups who were told about the 3 categories: effort-kinds and effort-order).

We also examined (for all groups of learners) whether declarative knowledge was related to performance on any of the forced-choice tests: word segmentation, order, or category structure. As shown in Figure S2, performance on word segmentation is significantly correlated with the number of words (*r*=.256, *p*=.042) that learners correctly produce (this was not true for the number of correct syllables (*r*=.191, *p*=.135). Interestingly, this declarative knowledge is not related to performance on measures of order (words: *r*=.230, *p*=.070; syllables: *r*=.031, *p*=.807) or any of the category structure sub-tests (novel-with-TP, words: *r*=.107, *p*=.405, syllables: *r*=.070, *p*=.584; novel-no-TP, words: *r*=-.082, *p*=.522, syllables: *r*=-.238, *p*=.060; novel-good-vs.-bad, words: *r*=-.062, *p*=.627, syllables: *r*=-.039, *p*=.760), suggesting that declarative knowledge is related to word-level TP knowledge, but not other—higher order—aspects of the language. Notice that the correlation between the number of words produced and the category order test has the lowest of the non-significant p values (p=.070). One possible interpretation of this is that learning of the words and word order is related. Perhaps once leaners have a representation of words, they can then start to extract the across word TPs to learn the order.

*References*

1. Katz J (2005) SoftVoice (Demo Program). Los Angeles, CA: SoftVoice, Inc.

2. Schneider W, Eschman A, Zuccolotto A (2002) E-Prime user’s guide. Pittsburgh, PA: Psychology Software Tools.

*Footnotes*

1. These declarative data were collected from all participants in the effort words and effort kinds conditions and a subset of participants in the no effort condition (Experiment 1; 6 of 22) and effort order conditions (17 of 22).

*Figure Captions*

*Figure S1.* Average performance on each word in Experiment 1 (a) and Experiments 1 and 2 (b). Error bars reflect standard error of the mean. The dotted line reflects chance performance.

*Figure S2.* The number of words (a) and syllables (b) plotted against performance on the word segmentation test. Data are reported across all four groups of learners.
